# Minimally invasive unilateral pedicle screws and a translaminar facet screw fixation and interbody fusion for treatment of single-segment lower lumbar vertebral disease: surgical technique and preliminary clinical results

**DOI:** 10.1186/s13018-017-0606-z

**Published:** 2017-07-20

**Authors:** Peng Huang, Yiguo Wang, Jiao Xu, Bo Xiao, Jianheng Liu, Luyang Che, Keya Mao

**Affiliations:** Department of Orthopaedics, Chinese PLA General Hospital, Beijing, 100853 China

**Keywords:** Minimally invasive spinal surgery, Translaminar facet screw fixation, Pedicle screw fixation, Lumbar degenerative disease

## Abstract

**Background:**

Conventional open transforaminal lumbar interbody fusion (TLIF) using unilateral pedicle screws and a translaminar facet screw has been performed for many years with good results. The outcomes of minimally invasive TLIF (MIS TLIF) are similar to the good outcomes of open TLIF, with the additional benefits of reducing iatrogenic injury, shortening hospital stays, and reducing the recovery duration. Instead of using small cuts on both sides, we performed MIS TLIF through a single cut using unilateral pedicle screws and a translaminar facet screw. The operative feasibility, efficacy safety, and benefits of single-level MIS TLIF of such techniques require further clarification.

**Methods:**

A total of 60 patients with various single-segment lower lumbar vertebral diseases were treated in our department from January 2010 to March 2013. All the patients were initially performed single-level MIS TLIF using a hybrid construction of unilateral pedicle screws and a translaminar facet screw. Patient demographics and operative data were collected. The clinical outcomes were assessed before surgery and 3, 6, 12, and 24 months after surgery using the visual analog scale (VAS) for back and leg pain and the Oswestry Disability Index (ODI). Radiologic assessment of the lumbar spine with static and dynamic plain radiographs was performed 3, 6, 12, and 24 months after surgery. The fusion rates were assessed by an independent radiologist 2 years after surgery according to the Bridwell interbody fusion grading system.

**Results:**

No patients experienced significant postoperative complications. Excepting two cases, 58 cases were followed up for 24–38 months, averaged 29.9 ± 4.1 months. The patients’ average age was 46.6 ± 11.5 years, operative time 109.7 ± 17.8 min, intraoperative blood loss 67.3 ± 29.7 ml, length of incision 29.0 ± 3.2 mm, fluoroscopy time 31.1 ± 7.2 s, time to ambulation 20.3 ± 7.0 h, length of hospital stay 5.1 ± 1.1 days, and length of the translaminar facet screw 51.7 ± 3.4 mm. Screw position results: type I, 54 cases with 54 segments; type II, four cases with four segments. There were two (3.4%) translaminar facet screw failures, which were intraoperatively converted to a bilateral pedicle screw fixation procedure and excluded from the research. The postoperative images showed good positioning of the hybrid internal fixation, and all of the translaminar facet screws penetrated the facet joint. Two (3.6%) translaminar facet screws penetrated the lateral lamina and two (3.6%) translaminar facet screws penetrated the medial lamina without any serious neural complications. During the follow-up, there was no screw loosening or pedicle fracture observed. The VAS and ODI scores were significantly improved compared with the preoperative scores (*P* < 0.05), and the symptoms disappeared gradually. Fifty-one patients (87.9%) achieved grade I fusion radiographically at the final follow-up.

**Conclusions:**

MIS TLIF using a hybrid construction of unilateral pedicle screws and a translaminar facet screw is safe and effective in the treatment of single-segment lower lumbar vertebral disease, and it can be used as an optimal choice for fixation and fusion of some single-segment lower lumbar vertebral diseases.

## Background

Bilateral pedicle screw fixation combined with interbody fusion has been recognized as the “gold standard” treatment for the lumbar vertebral disease, which has a variety of advantages, such as great fixation intensity, excellent stability, and high fusion rate [1–8]. However, with its extensive use in the clinic, many disadvantages of the treatment have been reported, which includes long skin incision, considerable stress of internal fixation by strong fixation, stress shielding in the fixed segment, and the potential accelerated degeneration of adjacent segments [9–11]. Thus, surgeons have searched several modified fixation techniques, such as unilateral pedicle screw (UPS), and UPS plus contralateral translaminar facet screw (UPSFS) which has come into use, and acquired good clinical outcome and satisfied fusion rate [12–14].

Unilateral pedicle screw fixation combined with interbody fusion has been extensively used in the clinic, which has gotten a primarily good clinic result. Significant reductions in operation time, duration of hospitalization, and costs have been cited as the benefits of unilateral pedicle screw fixation (PSF) [12]. And some studies even showed that the unilateral PSF has equivalent fusion rates compared with the bilateral PSF [15]. However, biomedical studies indicated that this method failed to control lateral bending and resist torsional forces, potentially resulting in stress concentration and increasing the risk of internal fixation failure [16–19]. Translaminar facet screw fixation is another important method for lumbar fixation. Many relevant studies confirmed its availability in the clinic application [20, 21]. The translaminar facet screw was first introduced by King in 1948, and this technique involved the insertion of a short screw across the facet joint [22]. Translaminar facet screw fixation (TFSF) was initially described as a form of posterior instrumented fusion for lumbosacral degenerative disease by Magerl in the 1980s [23, 24]. The screw is a long screw that enters through the base of the spinous process on one side, fixes the contralateral facet joint after traversing the lamina, and ends at the base of the transverse process [23]. This procedure has been shown to be a successful technique that offers ease of procedure, smaller incisions, few complications, and reduced implant costs [25–29].

In addition to interbody fusion, TFSF offers a strong alternative to PSF with the same indications. However, the traditional use of TFSF also requires extensive paraspinal muscle retraction for insertion with consequent increased infection rates and muscle injury, and it carries a risk of neural and vascular damage as a result of improperly placed screws [7, 26, 30]. Researchers have attempted to achieve satisfactory lumbar fusion using minimally invasive (MIS) techniques that reduce injury and implant costs.

As a means of providing suitable spine stiffness with minimal injury and implant load, a hybrid construction of unilateral pedicle screws with a contralateral translaminar facet screw has been studied more frequently [14, 18, 31–34]. Biomechanical studies have certified the comparable strengths of bilateral PSF and unilateral pedicle screws and translaminar facet screw combination [18, 31, 33, 34]. Clinical outcomes have shown that open transforaminal lumbar interbody fusion (TLIF) using unilateral pedicle screws and a translaminar facet screw offers good results [14]. However, few studies on MIS TLIF using unilateral pedicle screws and a translaminar facet screw have been reported [32]. Hence, the feasibility, clinical outcomes, and fusion rates of unilateral pedicle screws combined with a translaminar facet screw in single-segment lower lumbar vertebral disease were investigated in this study.

## Methods

### Ethics statement

This study has been approved by the Ethics Committee of the Chinese PLA General Hospital, and the approval number is K2010-011-02. Written informed consent was obtained from each patient prior to the study.

### Inclusion and exclusion criteria

Inclusion criteria were patients with (I) single-segment lower lumbar vertebral disease; (II) chronic low back pain with or without neurological symptoms of lower extremities; and (III) inefficacy after strict conservative treatment for more than 6 months. Imaging findings showed serious single segmental degeneration disease, unilateral intervertebral disc herniation, or lumbar instability, which are consistent with the symptoms and signs. Exclusion criteria were (I) lumbar degenerative spondylolisthesis (II degree or higher); (II) lumbar spondylolysis; (III) serious three-dimensional deformity of lumbar vertebrae; (IV) obvious osteoporosis of lumbar vertebrae; and (V) patients with multi-segment lumbar degenerative disease, revision surgery, spinal tumor, acute spinal trauma, and spinal infections.

### General information

This study included 60 patients (34 male, 26 female) aged 22 to 67 years (mean, 46.6 years). All the patients have a history of lumbar degenerative disease and had been treated conservatively for at least 6 months without success. The patients were evaluated with a routine lumbar X-ray, computed tomography (CT), and magnetic resonance imaging at admission, and the signs and symptoms of the patients were consistent with the imaging findings. Fifty-eight patients underwent single-level MIS TLIF by the same experienced surgeon using a hybrid construct fixation with unilateral pedicle screws combined with contralateral translaminar facet screw. It was the first lumbar surgery at that level for all of the patients, and the indication for surgery was chronic low back pain, intermittent claudication, and unilateral radicular complaints. The translaminar facet screw length and the thickness and oblique angle of the laminar were measured according to the preoperative lumbar X-ray and CT. The hybrid construct fixation was used at the L4–L5 level in 23 patients and at the L5–S1 level in 35 patients. Thirty-five patients underwent translaminar fixation on the left side, and 23 patients underwent fixation on the right side. The minimum follow-up was 24 months (range 24 to 38 months).

### Surgical technique

**Fig. 1:**
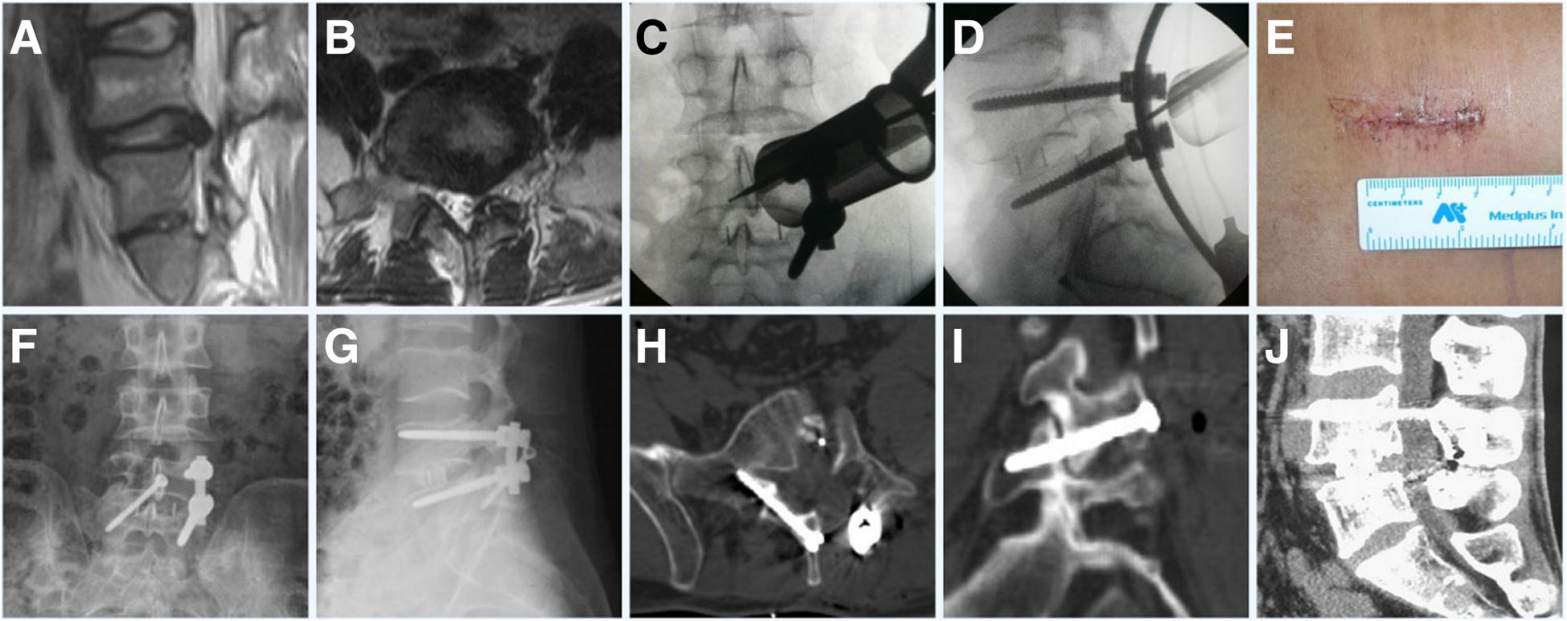
The preoperative MRI (**a**, **b**) showed a herniated disc. Accurate placement of the translaminar facet screw (**c**, **d**) was accomplished using the C arm intraoperatively. A small skin incision (**e**) was showed. Anteroposterior (**f**) and lateral (**g**) views after MIS TLIF showed good position of the hybrid internal fixation. CT scans (**h**–**j**) at 2 years after surgery demonstrated solid interbody fusion, and the translaminar facet screw entered through the base of spinous process on one side, fixed the contralateral facet joint after traversing the laminar, and ended at the base of the transverse process

The MIS TLIF procedure was performed on the side that appeared symptomatic. Under general anesthesia, the patient was placed in a prone position on a radiolucent operative frame. The surgical procedure consisted of the following steps:

1. Incision and placement of tubular retractor: With the help of a C arm, a longitudinal incision was made in the skin 3–4 cm lateral to the midline on the symptomatic side. The incision was generally 2.5–3 cm long, which was sufficient for the placement of the tubular retractor (METRx system, Medtronic Sofamor Danek, USA).
2. Discectomy, decompression, and fusion: After blunt dissection of the longissimus and multifidus muscles, progressive dilation of the dissected plane was completed. The tubular retractor was then docked over the facet joint at the level of the surgery. After clearance of the reliquus soft tissues under direct vision, the peripheral lamina and the facet joint were exposed. Canals for the pedicle screws were prepared with the help of a C arm. A complete facetectomy was then performed, and the ligamentum flavum was resected. After the traversing and exiting nerve roots were identified, a rigorous discectomy was conducted and the cartilage end plates were removed using curettement. A sufficient local autologous bone graft taken from the removed facet was used to fill the disc space. A single appropriately sized PEEK cage (Concorde, DePuy Spine, USA) packed with locally harvested autologous cancellous bone was inserted obliquely across the disc space. When sufficient decompression was completed, two pedicle screws and a rod (Exp, Depuy Spine, USA) were implanted before the final tightening.
3. Translaminar facet screw fixation: The lateral and laminar angles of the translaminar facet screw to be fused were measured preoperatively using X-ray and CT or MR imaging. After the MIS TLIF and unilateral PSF procedure, the tube was adjusted to provide an oblique view of the base of the superior spinous process. This facilitated the entry of a 2.2-mm-diameter drill. Considering the preoperatively determined lateral and laminar angles, the drill was inserted through the same incision under radiological guidance. During the drill’s insertion, care was taken to ensure that it remained within the cortical confines of the lamina. After drilling, a 4.5-mm-diameter cortical screw (AO Spine, USA) of suitable length (usually 45–58 mm) was inserted for fixation, traversing through the lamina and facet joints and terminating at the base of the transverse process. Accurate placement of the screw was confirmed using the C arm or intraoperative CT prior to wound closure. A representative case is presented in Fig. 1.

### Postoperative treatment

Patients received routine postoperative management, including infection prevention, and low-dose hormones and correction of dehydration and gastric mucosal protective measures and bed rest. After waking from anesthesia, the patients were encouraged to actively dorsiflex the ankle and perform straight leg raises with both lower legs. All the patients did not need to have a drainage tube. Patients wore waist support and were encouraged out-of-bed activity 3 to 5 days postoperatively. With the help of a waist girdle, progressive back and abdominal muscle exercises were initiated at the sixth week postoperatively.

### Evaluation method

The data recorded for analysis were age, gender, operative time, intraoperative blood loss, length of incision, X-ray exposure time, length of the translaminar facet screw, time to ambulation, length of hospital stay, complications, and the clinical and radiographic results after surgery. After surgery or discharge from the hospital, the patients received regular, close follow-up (at 3 days, 3 months, 6 months, and 1 year postoperation and annually thereafter). Clinical and radiological evaluations were conducted at every follow-up visit. All of the data were collected prospectively after a minimum of 2 years of follow-up. The patients were evaluated using the visual analog scale (VAS) for leg and back pain and the Oswestry Disability Index (ODI), version 2.0. The radiological evaluation included anteroposterior, lateral, oblique, and flexion-extension plain radiography, CT scans, and MRI. Translaminar facet screw and interbody fusion were assessed by imaging. Translaminar facet screw position was classified into three types: type I, the translaminar facet screw is located in the lamina; type II, the translaminar facet screw penetrates the lamina partially; and type III, the translaminar facet screw penetrated the lamina completely [35]. Fusion rates based on the Bridwell interbody fusion grading system were assessed using static and dynamic plain radiography 2 years postoperatively [36]. The Bridwell interbody fusion grading system is provided in Table 1.

**Table 1:** Bridwell interbody fusion grading system

Grade	Description
I	Fused with remodeling and trabeculae present
II	Graft intact, not fully remodeled and incorporated, but no lucency present
III	Graft intact, potential lucency present at top and bottom of graft
IV	Fusion absent with collapse/resorption of graft

### Statistical analysis

SPSS 17.0 (SPSS, Chicago, IL) was used for the statistical analyses. Normally distributed continuous variables are shown as the mean ± SD. Analysis of variance (ANOVA) was performed for the VAS and ODI scores. A *P* value less than 0.05 was considered statistically significant.

## Results

### General information

A total of 58 patients were followed up in the study. The mean age of the patients was 46.6 ± 11.5 years (range 22–67 years), and the mean follow-up period was 29.9 ± 4.1 months (range 24–38 months).

### Operative data

The mean operative time was 109.7 ± 17.8 min (range 73–157 min); the mean intraoperative blood loss was 67.3 ± 29.7 ml (range 26–157 ml); the mean length of incision was 29.0 ± 3.2 mm (range 24–37 mm); the mean fluoroscopy time was 31.1 ± 7.2 s (range 19–53 s); the mean ambulation time was 20.3 ± 7.0 h (range 8–42 h); the mean length of hospital stay was 5.1 ± 1.1 days (range 3–8 days); and the mean translaminar facet screw length was 51.7 ± 3.4 mm (range 45–58 mm). Translaminar facet screw positions were assessed as follows: type I, 54 cases with 54 segments, and type II, four cases with four segments.

### Follow-up

During the follow-up, there were significant improvements in both the VAS score for back and leg pain and the ODI scores at all time points compared with the preoperation scores (*P* < 0.05). The symptoms disappeared gradually. The VAS and ODI scores are listed in Table 2. Fifty-one patients (87.9%) achieved grade I fusion radiographically at the 2-year follow-up. There were no cases of grade III or IV fusions.

**Table 2:** Results of VAS and ODI score

	VAS back	VAS leg	ODI
Preoperative	6.2 ± 1.8	6.8 ± 1.8	57.7 ± 15.5
Three days after operation	3.1 ± 1.5^*^	1.7 ± 1.2^*^	-
Three months after operation	1.8 ± 1.2^*#^	1.4 ± 1.2^*^	27.6 ± 11.1^*^
Six months after operation	1.6 ± 1.3^*#^	1.4 ± 1.3^*^	25.1 ± 8.8^*^
One year after operation Two years after operation	1.4 ± 1.4^*#^ 1.1 ± 0.9^*#△^	1.2 ± 1.0^*#^ 0.9 ± 0.8^*#△▽^	19.1 ± 8.6^*△▽^ 13.8 ± 5.8^*△▽⊿^
*P*	0.000	0.000	0.000

Note: “-” stands for no data

*compared with preoperative value, *P* < 0.05; #compared with the value at 3 days after operation, *P* < 0.05; △compared with the value at 3 months after operation, *P* < 0.05; ▽compared with the value at 6 months after operation, *P* < 0.05; ⊿compared with the value at 1 year after operation, *P* < 0.05

### Complications

Two (3.4%) cases of small dural tears during decompression were not repaired, and these two patients were kept on bed rest for 10 days with no subsequent postoperative cerebrospinal fluid leakage. One (1.7%) case of fat liquefaction experienced primary healing after physical therapy and changing of the dressings. There were two translaminar facet screw failures that were intraoperatively converted to pedicle screws (bilateral PSF). The postoperative images showed good positioning of the hybrid internal fixation, and all of the translaminar facet screws penetrated the facet joint. Two (3.6%) translaminar facet screws penetrated the lateral lamina, and two (3.6%) translaminar facet screws penetrated the medial lamina with no serious neural complications. One (1.7%) patient who required temporary new radiculopathy because of translaminar facet screw malpositioning experienced a full recovery within 2 weeks after subsequent corrective surgery.

## Discussion

In this study, a tubular retractor was adopted to expose the lamina and articular process. Facetectomy, discectomy, intervertebral clearance, intervertebral bone grafting, and cage placement were then performed. Unilateral pedicle screws combined with a contralateral translaminar facet screw fixation based on preoperative measured data were conducted. Previous studies indicated that translaminar facet screw fixation is a simple, safe, and satisfactory method [29, 37]. Our results revealed that the direction of the drill in the lamina and screw placement were the most important step in this operation. Postoperatively, imaging data indicated that the position of the translaminar facet screws was good (type I, 53 cases with 54 segments; type II, three cases with four segments). In this operation, only a 3–4-cm longitudinal incision was made in the skin lateral to the midline on the symptomatic side, while the muscles, facet joint, and laminar on the contralateral side remained intact, which is important to reduce surgical trauma and blood loss and shorten operative time [38]. The mean operative time was 109.7 ± 17.8 min (range 73–157 min); the mean intraoperative blood loss was 67.3 ± 29.7 ml (range 26–157 ml); the mean length of incision was 29.0 ± 3.2 mm (range 24–37 mm); the mean fluoroscopy time was 31.1 ± 7.2 s (range 19–53 s); the mean ambulation time was 20.3 ± 7.0 h (range 8–42 h); the mean length of hospital stay was 5.1 ± 1.1 days (range 3–8 days); and the mean translaminar facet screw length was 51.7 ± 3.4 mm (range 45–58 mm). The low intraoperative blood loss, small skin incision, reduced trauma, and short operative time all contributed to moderate pain and rapid recovery postoperatively. In addition, all patients wore waist support and were encouraged out-of-bed activity 3 to 5 days postoperatively. With the help of a waist girdle, progressive back and abdominal muscle exercises were initiated at the sixth week postoperatively and no fixation loosening and breakage were observed during the follow-up. Fifty-one patients (87.9%) achieved grade I fusion radiographically at the 2-year follow-up. There were no cases of grade III or IV fusions. Sethi et al. also achieved bony fusion among 19 patients with low lumbar lesions using unilateral pedicle screws and a translaminar screw fixation technique [14]. The results of Sethi and our study both indicated that unilateral pedicle screw fixation combined with contralateral translaminar facet screw fixation and interbody fusion technique can obtain satisfactory clinical results. Moreover, significant differences in VAS and ODI scores were observed between the final follow-up and preoperation. The leg and back pain, lumbar function, and activities of daily living were obviously improved. Compared with the conventional internal fixation technique [14, 39], our study indicated that unilateral pedicle screw fixation combined with contralateral translaminar facet screw fixation and interbody fusion can obtain the same satisfactory clinical results.

However, several other surgical techniques are available to obtain single segmental lumbar interbody fusion at present. PSF is frequently used to provide temporary spinal stability after spinal surgery until a fusion mass forms [12, 15]. Although it offers the advantage of solid stability, bilateral PSF use is associated with higher implant costs and an increased incidence of neurologic complications [40, 41]. Furthermore, it requires extensive paraspinal muscle retraction for the insertion of the screws, which results in increased rates of infection and muscle injury, and improperly placed screws can lead to neural and vascular damage [7, 26, 30]. With the aim of reducing operative time and implant costs, some researchers have attempted to use unilateral PSF for lumbar spinal fusion [12]. However, unilateral PSF has not been recommended for long fusions despite studies that show comparable fusion rates for unilateral and bilateral PSF [15, 18]. The authors of several biomechanical investigations have demonstrated that unilateral PSF decreases spinal stiffness [18, 19].

Many researchers have shown that translaminar facet screw insertion provides a comparable rate of fusion and satisfactory clinical outcomes provided that the indications are correctly applied [29, 37]. The current study supports the use of TFSF for short-segment fusion in the lumbar spine as a successful technique with the benefits of a relatively simple procedure, smaller incisions, few complications, and reduced implant costs [25–29]. Currently, the hybrid construct of unilateral pedicle screws combined with a contralateral translaminar facet screw is being increasingly investigated because it provides suitable spine stiffness with minimal injury and implant loads [14, 18, 31–34]. Several biomechanical studies have shown that the hybrid construction provides a strength similar to that of bilateral PSF [18, 31, 33, 34]. Sethi and Lee documented the good clinical outcomes of open TLIF using unilateral pedicle screws and a translaminar facet screw (similar to the system we used in our study), and they suggested that the hybrid construction offered a less expensive and more viable option for single-level lumbar fusion [14]. Jang and Lee published their pilot clinical studies of the unilateral pedicle screw (PS)-based and contralateral facet screw (FS)-based TLIF techniques, and they indicated that TLIF with ipsilateral PS and contralateral FS fixation offered reduced blood loss and soft-tissue injury compared with the conventional TLIF [32].

Researchers have attempted to achieve satisfactory lumbar fusion with MIS techniques while reducing injury and implant costs [42]. The morbidity associated with open TLIF is extensive, and prolonged muscle ischemia occurs as a result of the extensive muscle stripping and retraction that occurs during the surgical approach [43, 44]. The MIS TLIF procedure is used to achieve solid lumbar interbody fusion via a unilateral posterolateral approach, and it has gained recent popularity because it results in smaller wounds, less tissue trauma, and faster recovery [45, 46]. However, wide exposure and expensive implants are required for the insertion of the percutaneous pedicle screws in standard MIS TLIF [14, 32]. In addition, the soft-tissue injuries caused by the insertion of percutaneous pedicle screws include damage to muscles, the adjacent facet, and ligaments. The procedure can cause increased blood loss, infection rate, and postoperative back pain; a longer recovery period; and impaired fusion [30, 44, 47].

For the disadvantages previously mentioned above, we performed MIS TLIF in 58 patients using a hybrid internal fixation system consisting of unilateral pedicle screws and a contralateral translaminar facet screw. The technique of MIS TLIF used in this study was quite different from the standard MIS TLIF [14, 32], and our result revealed that it is characterized by a small incision, reduced trauma, simple operation, high safety, good stability, high fusion rate, and few complications. And we determined that it is feasible and safe to insert a translaminar facet screw under direct vision via a single small incision and that there was no decrease in stiffness or effectiveness compared with the conventional bilateral PSF procedure. Because the anatomical structures surrounding the canal created for the translaminar facet screw include the posterior muscle, the anterior ligamentum flavum, and the superior and inferior pedicle cortical bone, there is a relatively extensive safe area for the insertion of the translaminar facet screw. This safety statement was also justified by the fact that in our study, two (3.6%) translaminar facet screws penetrated the lateral lamina and two (3.6%) translaminar facet screws penetrated the medial lamina with no serious neural complications. Additionally, there was a relatively steep learning curve for this technique.

However, there are several limitations to this current study. Firstly, it is a retrospective investigation, which cannot avoid selection and recall bias completely, despite our trying our best to collect and analyze the data meticulously throughout the study. Secondly, the patients with bilateral radicular symptoms were not included. In fact, our group has already adopted this technique to treat patients with bilateral symptoms and two segment degenerative lumbar disease. Next, we will report the preliminary result of this aspect of the research. Last, but not the least, the sample size in this study was relatively small and the follow-up time of 29.9 months was relatively short to observe the long-term clinical outcome. And it is just a preliminary clinical study about the feasibility of this surgical technique and a clinical result. In the future, well-designed prospective studies with a larger study population and longer follow-up time should be conducted to determine the clinical and radiographic significance of the MIS TLIF technique compared with bilateral PSF technique and other internal fixation methods, providing a convincing evidence-based conclusion.

## Conclusion

We have introduced the MIS TLIF technique using a hybrid internal fixation system constructed with unilateral pedicle screws and a contralateral translaminar facet screw which was different from the conventional MIS TLIF. The MIS TLIF technique is safe and effective in the treatment of single-segment lower lumbar vertebral disease, and it can be used as an optimal choice for the fixation and fusion of some single-segment lower lumbar vertebral diseases.

## Abbreviations

CT: Computed tomography
ODI: Oswestry Disability Index
PSF: Pedicle screw fixation
TFSF: Translaminar facet screw fixation
TLIF: Transforaminal lumbar interbody fusion
VAS: Visual analog scale
